# Association between Family and School Pressures, Consumption of Ultra-Processed Beverages, and Obesity in Preadolescents: A School-Based Epidemiological Study

**DOI:** 10.3390/children10030500

**Published:** 2023-03-02

**Authors:** Ioannis Gketsios, Thomas Tsiampalis, Alexandra Foscolou, Ioanna Panagiota Kalafati, Tonia Vassilakou, Aikaterini Kanellopoulou, Venetia Notara, George Antonogeorgos, Andrea Paola Rojas-Gil, Odysseas Androutsos, Ekaterina N. Kornilaki, Areti Lagiou, Demosthenes B. Panagiotakos, Rena I. Kosti

**Affiliations:** 1 Department of Nutrition and Dietetics, School of Physical Education, Sports and Dietetics, University of Thessaly, 42132 Trikala, Greece; 2 Department of Nutrition and Dietetics, School of Health Science and Education, Harokopio University, 17671 Athens, Greece; 3 Department of Public Health Policy, School of Public Health, University of West Attica, 11521 Athens, Greece; 4 Department of Public and Community Health, Laboratory of Hygiene and Epidemiology, School of Public Health, University of West Attica, 11521 Athens, Greece; 5 Department of Nursing, Faculty of Health Sciences, University of Peloponnese, 22100 Tripoli, Greece; 6 Department of Preschool Education, School of Education, University of Crete, 74100 Rethymnon, Greece; 7 Faculty of Health, University of Canberra, Bruce, ACT 2617, Australia

**Keywords:** soft drinks, flavored drinks, preadolescents, children, stress, obesity, body mass index

## Abstract

The aim of the present work was two-fold. Firstly, to evaluate the association between the consumption of ultra-processed beverages (UPB) on preadolescents’ likelihood of being obese. Secondly, to investigate the potential impact of family and school environmental stressors on this unhealthy lifestyle habit. A cross-sectional study was conducted among 1718 Greek preadolescents and their parents, during the school years 2014 to 2016. Parental and child characteristics were collected anonymously, through self-administered and validated questionnaires. Among others, UPB consumption (soft and flavored drinks) was recorded, classifying children as low, moderate, or high consumers, while anthropometric characteristics [height, body weight, Body Mass Index (BMI)] were also recorded. Almost seven out of ten preadolescents were classified as at least moderate UPB consumers, while approximately three out of ten were classified as high UPB consumers. Higher UPB consumption was associated with significantly higher levels of BMI, while preadolescents living in a more stressful family and school environment were found to consume significantly higher amounts of UPB. Stakeholders should implement programs that raise awareness among parents and teachers about the sources of stress in preadolescence as a potential “triggering factor” of unhealthy dietary preferences.

## 1. Introduction

Childhood and preadolescence obesity remains a serious global burden, despite public health initiatives [1]. Due to the multifactorial nature of obesity, its prevention and treatment are still unsolved [2]. As several researchers have already demonstrated, a sedentary lifestyle and the consumption of “energy-dense” foods and soft drinks with added sugar, rather than purely endocrinological or genetic factors, are the main environmental causes of the obesity epidemic, even in early childhood [3,4]. It is worth mentioning that the consequences of childhood obesity are tracking into adulthood and increasing the risk of obesity, cardiovascular diseases, type II diabetes mellitus, and premature mortality in later life [1,2].

Soft drinks, i.e., nonalcoholic beverages, frequently carbonated, that contain natural or artificial sweeteners and flavors, and sometimes juice [5], have been linked to the obesity pandemic [6]. However, it is not only soft drinks with added sugars that have been considered potentially harmful. Accordingly, even though there are no clear findings about the effects of concentrated fruit juices and chocolate milk on Body Mass Index (BMI) status [7,8,9], both of them are considered concentrated sources of sugar for children and have been accused of contributing extra calories due to the added sugars [10].

Indeed, in accordance with the recently published Position Paper of the European Academy of Paediatrics and the European Childhood Obesity Group, the consumption of sugar-sweetened beverages (SSB) has to be limited among children and adolescents [11] in an attempt to contain the burden of childhood obesity. Adolescents’ and children’s soft drink intake appears to be significantly influenced by factors such as gender, educational goals, dietary restrictions, ease of access, parental role models, adolescents’ attitudes, and preferences [12,13]. At the same time, stress has recently been revealed to be associated with children’s weight status [14]. Adolescence and preadolescence may be a vulnerable time for social stress [15], which has been related to functional and structural changes in brain systems, crucial for social-affective processing and cognitive regulation [16]. Potential environmental sources of stress among preadolescents may stem from anxiety due to conflict with parents, and peer relationships [17,18] as well as from school-related stress as a result of educational attainment [19]. The findings reported in the literature suggest a bidirectional association between SSB consumption and emotional distress. In particular, drinking SSB on a daily basis has been linked to increased anxiety and depression among youngsters [20], while it has also been suggested that children and adolescents may use soft drink consumption as a dysfunctional strategy for coping with mental health difficulties [21]. It is also known that the consumption of foods that are rich in sugar or fat has been found to distract from stress [22], and thus, alleviating perceived emotional stress [23]. It seems that there may be an interplay between stress, consumption of ultra-processed beverages (UPB), and the likelihood of obesity in preadolescence. However, there is scarce evidence regarding the dominant stressors in preadolescence and their association with UPB consumption. In this context, the hypothesis of the present study was that environmental stress affects UPB consumption in preadolescents, influencing the likelihood of being obese. Thus, the aim of the present work was double-fold: (1) to evaluate the association between the consumption of UPB on preadolescents’ likelihood of being obese; (2) to investigate the potential impact of family and school environmental stressors on this unhealthy lifestyle habit.

## 2. Materials and Methods

### 2.1. Design and Setting

This is a cross-sectional, school-based, observational study.

The study took place in the metropolitan Athens area, in the Heraklion city area (the capital city of the island of Crete), and in three counties of the Peloponnese peninsula (Sparta, Kalamata, and Pyrgos). The regions represent large urban and rural municipalities from southern Greece. The enrollment procedure was carried out during the school years 2014 to 2016. During the two consecutive school years, a gradual enrollment of schools was conducted. The schools that participated in this study were selected using random sampling from a list provided by the Greek Ministry of Education; all children aged 10–12 were then asked to participate. In total, 47 primary schools (32 from the Athens area, 5 from Heraklion, 3 from Pyrgos, 2 from Kalamata, and 5 from Sparta) were included. More information can be found elsewhere [24].

### 2.2. Bioethics

Before starting the study, approval was requested from the appropriate department of the Ministry of Education and Religious Affairs (code of approval F15/396/72005/C1 by the Institute of Educational Policy) and the study was carried out following the principles of the Declaration of Helsinki. The investigators informed all people who were involved about the aims and procedures of the research. The students participated in the study after the written consent of their parents.

### 2.3. Sample

Students and their parents were recruited through school registries. Children’s questionnaires were filled out in school settings, whereas parents’ questionnaires were filled out at home and returned to school. In total, 1728 students (795 males; 46%) aged 10–12 years old, enrolled in the study. The participation rate of children ranged from 95 to 100% between schools, without any significant differences between the studied areas. After checking the questionnaires’ completeness for the needs of the present work, the final working sample for the analyses was n = 1716 children. All children’s parents were also invited to participate, with a 68.9% response rate being achieved (n = 1190). The working sample was adequate to evaluate the effect size measures’ differences of 20% at <5% level of significance achieving 85% statistical power.

### 2.4. Measurements

#### 2.4.1. Children’s Characteristics

Each child completed a questionnaire specially developed for the study. To avoid errors and discrepancies, the study’s investigators assisted children by giving practical examples when it was necessary. Each child was provided with a personal code by the school principal for the questionnaires to be cross-referenced to those of their parents. The questionnaire retrieved information about socio-demographics (age, sex), anthropometric characteristics as well as dietary habits, and lifestyle parameters.

#### 2.4.2. Anthropometric Characteristics

Specially trained health scientists/investigators (i.e., dietitians, registered nurses, physicians) took the necessary anthropometric measurements of children (height and body weight in cm and kg, respectively) using a tape measure and a scale (with skin-tight clothing, to minimize measurement errors) and performed a face-to-face interview with them, which lasted a maximum of 10–15 min. Each child’s body weight (kg) was measured to the nearest 100 g using a digital scale (Tanita), and height (cm) was measured to the nearest 0.1 cm using a portable stadiometer (Leicester Height Measure). Children’s BMI was calculated as the ratio of kg/height in m^2^. Children’s body weight status was evaluated through the age- and the sex-specific International Obesity Task Force (IOTF) Body Mass Index cut-off criteria [25].

#### 2.4.3. Physical Activity Status

The standardized, validated, and reliable questionnaire Physical Activity and Lifestyle Questionnaire (PALQ) [26] was used to measure children’s physical activity status. The latter was defined as their participation in out-of-school activities such as sports club participation, playing with others, running, and swimming, on a daily or weekly basis.

#### 2.4.4. Stress

Children’s stress was assessed through self-report questions, which reflected five sources of stress: Parental expectations (yes/no); Teachers’ expectations (yes/no); School performance (yes/no); Busy schedule of extracurricular activities (yes/no); Pressure from classmates and friends (yes/no). These were based on the Adolescent Stress Questionnaire (ASQ) [27] translated and evaluated in several countries, including Greece [28].

#### 2.4.5. Dietary Habits

The level of adherence to the Mediterranean Diet was evaluated using a Mediterranean Diet quality index for children and adolescents (i.e., KIDMED) [29]. Dietary habits with a positive aspect to this dietary pattern scored +1; dietary habits with a negative association scored −1; and, dietary habits with a neutral association scored 0. The theoretical total score ranges from −4 to 12. Lower scores indicated low adherence to the Mediterranean Diet while higher scores indicated high adherence to the Mediterranean Diet.

To acquire information on children’s eating habits, we used a validated semi-quantitative food frequency questionnaire (FFQ) [30] which contained all foods and beverages (either sugar-sweetened, or with no added sugar, or sugar-free beverages), including soft drinks, (i.e., carbonated soft drinks, concentrated fruit drinks) and flavored drinks (i.e., chocolate milk) which are commonly consumed by the general child population. Henceforth, for ease of reference, soft drinks and flavored drinks, all of them either sugar-sweetened or sugar-free or with no added sugar, will be referred to as UPB. For the purpose of this study, 330 mL of drink consumption was considered as the standard portion size.

More specifically, information regarding UPB consumption was collected, based on a daily (1 time or more than 2 times per day), weekly (i.e., 1, 2–6 times per week), or monthly (i.e., 1–3 times per month, less than 1 time per month) basis. Thus, following the methodological approach of Naomi et al. (2022) [31], each drink item was assigned a score of 0–5 depending on the frequency of drink consumption (0 being never/rarely and 5 being 1 or more times per day) so that the UPB consumption measure ranged from 0 to 15 (0 being no UPB consumption). Particularly, UPB consumption was summarized into three categories for the analyses in the present study: “Low” when the frequency consumption was equal or less than 1 time per week; “Moderate” for 2–6 times per week; “High” for at least 1 time per day. The same categorization was also applied to the frequency of chocolate milk consumption.

### 2.5. Parental Characteristics

Several parental sociodemographic characteristics (age), anthropometric characteristics [body weight (kg) and height (m)], educational level (i.e., primary, secondary, higher), and financial characteristics (income status under or over 18.000 €/year), as well as lifestyle characteristics [smoking status (yes/no), physical activity status (not at all or at least 1–2 times per week)], were recorded by the children’s parents.

### 2.6. Dietary Characteristics

Similar to children’s dietary habits evaluation, parental dietary habits were assessed on a semi-quantitative, validated, and reproducible FFQ. Overall assessment of dietary habits was evaluated through a special diet score (MedDietScore, range 0–55), which assesses adherence to the Mediterranean dietary pattern [32]. Higher values on the score indicate greater adherence to this pattern and, consequently, healthier dietary habits. Parents whose score was ≤25 units were classified as being away from the Mediterranean Diet, while parents whose score was >25 units were classified as being close/very close to the Mediterranean Diet.

### 2.7. Statistical Analysis

Continuous characteristics are presented as mean ± standard deviation (SD), and categorical characteristics as relative frequencies (%). The One-way Analysis of Variance (ANOVA) was used in order to investigate the association between the continuous characteristics and the frequency of UPB consumption (Low, Moderate, High) (dependent variable), while Pearson’s Chi-square test was used in the case of the categorical characteristics. Normality of the continuous variables’ distribution was tested through graphical (histograms, PP- plots, QQ- plots) and statistical means (Shapiro–Wilk test). Multivariable linear regression analysis was implemented in order to evaluate the association between preadolescents’ UPB consumption and their BMI (dependent variable). In addition, multivariable logistic regression analysis was also performed in order to investigate the individual effect, as well as the combined effect, of different sources of stress on UPB and chocolate milk consumption.

In the case of linear regression analysis, the results are presented as unstandardized beta-coefficients and standard errors, while in the case of logistic regression analysis, the results are presented as Odds Ratios (OR) and 95% Confidence Intervals (CI). Both the beta-coefficients and the ORs, compare all the UPB and chocolate milk consumption categories, with each other. All the results are adjusted for various participants’ characteristics (i.e., children’s age, sex, KIDMED score, physical activity status as well as parents’ age, BMI, smoking status, physical activity status, educational level, income and MedDietScore). All statistical analyses were performed using IBM SPSS Statistics, version 29 (IBM Corp., Armonk, NY, USA) and significance level was set at a = 0.05.

## 3. Results

### 3.1. Profile of High UPB Consuming Preadolescents

Table 1 presents the characteristics of the preadolescents and their parents, both in the total sample, as well as separately, according to the frequency of UPB consumption. Almost seven out of 10 preadolescents (67.1%) were classified as at least moderate UPB consumers, while 27.6% of the total sample were classified as high UPB consumers. As depicted, boys vs. girls (*p* = 0.001), as well as preadolescents with higher body mass index (*p* = 0.001) and unhealthier lifestyle habits, both in terms of lower physical activity levels (*p* = 0.002), as well as in terms of lower diet quality (*p* < 0.001), were more likely to be high UPB consumers. As far as the parents’ characteristics are concerned, high UPB consuming preadolescents were more likely to have parents characterized by lower educational levels (*p* < 0.05 both for fathers and mothers) and income (*p* = 0.040), while at the same time they seemed to have parents with both unhealthier nutritional habits (*p* < 0.001), as well as lower physical activity levels (*p* = 0.030). Finally, regarding the influence of the stress levels on UPB consumption, preadolescents being influenced by more stressors were more likely to be high UPB consumers (*p* = 0.015), and in particular, a significantly higher percentage of the high UPB consumers were found to be stressed due to parental expectations (*p* = 0.023), teachers’ expectations (*p* = 0.021), as well as by the pressure coming from their classmates and friends (*p* = 0.038).

### 3.2. Impact of High UPB Consumption on Preadolescents’ Body Mass Index

After adjusting for several preadolescents and their parents’ characteristics, as presented in Table 2, higher UPB consumption was associated with significantly higher BMI among preadolescents. More specifically, based on the fully adjusted model (Model 7), when compared to low consumers, preadolescents with high UPB consumption were found to have 0.89 kg/m^2^ higher BMI (b = 0.89; se = 0.37; *p* = 0.020). In addition, high UPB consuming preadolescents were also found to have significantly higher BMI levels, even when compared to moderate UPB consumers (b = 0.69; se = 0.32; *p* = 0.030). Finally, it should be noted that there was no significant difference between low and moderate UPB consuming preadolescents.

### 3.3. Association of UPB Consumption with Stress Levels

Moreover, multivariable logistic regression analysis was also performed in order to evaluate the association between the preadolescents’ UPB consumption and their stress levels. As depicted in Table 3, preadolescents who report being affected by a greater number of stressors are significantly more likely to be high UPB consumers than low UPB consumers (per 1 stressor increment: OR = 1.11; 95% CI = 1.01–1.24), while at the same time, they were found to have higher odds of being high UPB consumers than moderate UPB consumers (per 1 stressor increment: OR = 1.09; 95% CI = 0.99–1.20), with the difference being of borderline significance (*p* < 0.10). More specifically, preadolescents reporting that they are being stressed by their parents’ expectations, had 32% higher odds of being high UPB consumers than low consumers (OR = 1.32; 95% CI= 1.01–1.73), while those being stressed by their teachers’ expectations also had 33% higher odds of being high UPB consumers than low UPB consumers (OR = 1.33; 95% CI = 1.03–1.73). As for the pressure from their classmates and friends, preadolescents reporting this source of stress, were found to have 43% higher odds of being high UPB consumers than low UPB consumers (OR = 1.43; 95% CI = 1.02–2.02), while they were also found to have approximately two times higher odds of being high UPB consumers than moderate UPB consumers (OR = 1.91; 95% CI = 1.36–2.69).

In addition, the combination of the sources of stress seemed to increase the preadolescents’ odds of being high rather than low UPB consumers, with those reporting being stressed both by their parents’ expectations and their classmates/friends, as well as those being stressed by their parents, teachers, and classmates, having approximately two times higher odds of being high rather than low UPB consumers. Furthermore, it was also found that preadolescents being stressed by the pressure from classmates/friends, in combination with the parental and the teacher’s expectations, had at least two times higher odds of being higher rather than moderate UPB consumers. Finally, when compared to preadolescents reporting no source of stress, those reporting being stressed by all factors (parents, teachers, and classmates/friends), had 2.25 times and approximately three times higher odds of being high rather than low UPB consumers (OR = 2.25; 95% CI = 0.97–5.19) and high rather than moderate UPB consumers (OR = 2.84; 95% CI = 1.25–6.46), respectively.

Regarding the association between the frequency of chocolate milk consumption and the sources of stress, as presented in Table 4, there was an indication (*p* < 0.10) that a higher number of stressors is related to more frequent consumption of chocolate milk, while it should also be noted that preadolescents reporting being stressed by their parents’ expectations, as well as those being stressed by their classmates and friends’ pressure, were found to have approximately two times higher odds of being high rather than low chocolate milk consumers, as well as high rather than moderate chocolate milk consumers (all *p*-values < 0.05). When combining the effect of the stressors, it is worth noting the fact that, compared to preadolescents being not stressed at all, those being stressed by all factors were found to have at least four times higher odds of being high rather than low chocolate milk consumers (*p* < 0.05), as well as high rather than moderate chocolate milk consumers (*p* < 0.05).

## 4. Discussion

As it was hypothesised, environmental stress affects UPB consumption in preadolescents, influencing the likelihood of being obese. To the best of our knowledge, the role of the family and school environmental stressors on the frequency of soft and flavored drink consumption (either sugary sweetened, or with no added sugar) has been less investigated. The main study findings showed that nearly three out of 10 Greek preadolescents consumed at least one carbonated soft drink, chocolate milk and/or concentrated fruit drink on a daily basis. Boys, those having unhealthier lifestyle habits and higher BMI, as well as preadolescents belonging in families with lower socioeconomic level (in terms of educational and income level) and unhealthier lifestyle, were more likely to consume UPB on a more frequent basis. Moreover, it was revealed that the more frequent the UPB consumption, the higher the BMI for preadolescents, with family, teachers and peers being recognized as the stressors with the greater impact on UPB consumption. In particular, it was found that a higher number of stressors is significantly related to higher odds of being a high UPB consumer. From the public health perspective, this observation is of utmost importance, revealing the role of stress in preadolescents as a potential “triggering factor” of unhealthy dietary habits with its subsequent effects on childhood obesity. In line with other studies, Greek preadolescent boys were found to be more susceptible to soft drinks and other less healthy beverages compared to girls [33,34]. It is supported that young girls tend to have healthier dietary habits than boys [35], and this could be partly attributed to the fact that boys are more exposed to food advertising, and their tastes are more influenced by this exposure, which coincides with male-dominant advertisement content [36]. Moreover, the literature findings suggest that physiological, psychological and sociocultural factors determine the associations between gender and dietary behaviors. In particular, research has shown that males are prone to preferring strong-tasting, sweet foods and are driven by the pleasure of consumption [37]. In line with the literature, the current work revealed that after adjusting for several confounding factors, higher UPB consumption is related to higher preadolescents’ BMI [38,39], while it is also worth noting the fact that the level of preadolescents’ adherence to the Mediterranean Diet did not counterbalance the impact of high UPB consumption on BMI. Despite the fact that the Mediterranean Diet has been shown to have great benefits, both on children’s weight [40,41] and on their future weight as adults [42], it has also been demonstrated that excessive and regular intake of soft drinks might negate this impact, as it has been claimed that only substituting soft drinks with water could make youngsters lose weight [43]. The positive association between the consumption of UPB and weight status has been attributed to the high added sugar content, low satiety, and insufficient compensation for overall energy [44,45]. Recent findings also reveal the potential role of high-fructose corn syrup drinks on metabolic dysregulation [46], and on the alteration of gut microbiota, inducing obesity in animal studies [47].

In accordance with other studies supporting the premise that increased soft drinks, energy drinks, and generally sweet drinks or sweet food intake, are associated with increased stress levels and vice versa [48,49], it was found that the higher the number of stressors, the higher the preadolescents’ likelihood of being high UPB consumers, highlighting the effect of stress on sugar cravings. It is also worth mentioning that the role of chocolate milk has been investigated separately in our attempt to differentiate its consumption from the other undoubtedly unhealthy beverages. Albeit the association of flavored milk consumption with obesity has been proven to be rather protective regarding weight status [24,50], one could speculate that the higher the stressors, the higher the flavored milk consumption, implying the desire of preadolescents for the taste of sugar in stressful situations.

Chronic stress has been found to be correlated with a preference for high-sugar foods [51]. Stress from school is often linked to stress from parents and/or teacher’s demands as a whole. The literature suggests that the parent-child relationship, referring to the unique and enduring bond between a caregiver and his or her child, is one of the crucial factors which may influence students’ academic pressure [52]. Moreover, teachers’ behaviors and demands may affect students’ academic achievement and the level of school stress [53]. Preadolescents appraise their family and school environment as important for their well-being and struggle to cope with their excessive academic expectations, pleasing teachers and parents, and keeping up with their peers, which are all interrelated factors [54]. Evidence from the literature demonstrates that peer pressure is an influential force during adolescence, a period of special vulnerability, shaping both adaptive and maladaptive behaviors [55]. Moreover, parental expectations of their children’s academic performance compared to their peers, as well as the emphasis on family sacrifices to support their studies can serve as sources of stress and depression [56]. This is also observed in the present study implying that the demanding expectations of parents on their children’s academic performance are part of the Greek culture. Evidence suggests that the impact of stress on unhealthy eating may begin as early as in preadolescence [57]. A plausible speculation for the observed associations could be that overall, sugar-sweetened drinks increase weight gain by adding liquid calories to the diet, inducing hyperinsulinemia, as well as through a possible dopaminergic reward activation [6]. Additionally, cortisol is produced in response to stress [58] which in turn may induce cravings for sugary, fatty, and salty foods [59]. Stress also induces secretion of both glucocorticoids, and insulin which in turn increases motivation for food, and promotes food intake and obesity, respectively [60]. It is worth mentioning that the emerging concept of “depreobesity”, which incorporates the co-existence of obesity and depression, has been suggested to be the future epidemic [61].

From a public health perspective, numerous public health regulations such as taxation, marketing regulation, nutrition labeling, consumers’ education, and healthier food environments [62,63] have been designed to discourage youth and children from drinking unhealthy beverages. However, it seems that the roles of the family and the school environment have probably been underestimated regarding their influence on UPB consumption through the modulation of induced stress.

To the best of our knowledge, this is the first study in Greece, evaluating the effect of preadolescents’ sources of stress on the association between soft and flavored drink consumption with the BMI of Greek preadolescents. The novel findings, the large sample size with the implemented stratified random sampling scheme as well as the use of validated questionnaires, are considered the main strengths of the current work.

However, given our study’s cross-sectional design, numerous limitations should be addressed when interpreting the data. No temporal relationship and causal inferences can be established in observational studies, while self-reported questionnaires by schoolchildren may introduce reporting bias. Moreover, residual confounding may also exist. To eliminate this form of bias and maximize the validity of the replies, trained investigators were present during the entire process of completing the questionnaire in schools in order to clarify any potential misunderstandings.

## 5. Conclusions

Family, through parents’ expectations, and school stressors, either derived from teacher’s expectations or pressure from peers, seem to encourage UPB consumption which in turn contributes to an increased BMI in Greek preadolescents. It is thus urgent to identify and manage the interplay between stress, depression, unhealthy dietary habits and obesity so as to battle the epidemic of “depreobesity” in preadolescence. Stakeholders should implement programs that raise awareness among parents and teachers about the sources of stress in preadolescence as a potential “triggering factor” of unhealthy dietary preferences.

## Figures and Tables

**Table 1:** Preadolescents and their parents’ characteristics, both in the total sample, as well as stratified by the frequency of UPB consumption.

	Overall Sample (N = 1716)	Categories of Preadolescents’ UPB Consumption			*p*-Value
		Low (N = 565)	Moderate (N = 678)	High (N = 473)	
Children’s characteristics					
Age (in years)	11.2 ± 0.8	11.1 ± 08	11.2 ± 0.8	11.3 ± 0.8	0.065
Sex (% Boys)	46	43	43.7	53.1	0.001
Body Mass Index (in kg/m^2^)	19.2 ± 3.4	19.3 ± 3.4	19.1 ± 3.3	19.5 ± 3.6	0.001
Physical activity (% yes)	78.7	83.7	77.4	74.8	0.002
KIDMED score (−4 to 12)	5.0 ± 2.0	5.0 ± 2.3	4.6 ± 2.1	4.2 ± 2.5	<0.001
Parents’ characteristics					
Father’s age (in years)	45.8 ± 5.2	45.6 ± 5.1	46.2 ± 5.2	45.4 ± 5.3	0.100
Mother’s age (in years)	41.5 ±4.4	41.5 ± 4.1	41.6 ± 4.4	41.2 ± 4.7	0.480
Father’s educational level (% Higher education)	39.9	42.3	41.6	34.2	0.049
Mother’s educational level (% Higher education)	45.1	47.6	48.3	36.6	0.004
Income status (% >18,000 euros/year)	50.5	50.7	54.2	44.3	0.040
Father’s Body Mass Index (kg/m^2^)	27 ± 3.7	27 ± 3.8	26.8 ± 3.5	27.2 ± 3.9	0.350
Mother’s Body Mass Index (kg/m^2^)	24 ± 4.0	24 ± 4.3	23.8 ± 3.6	24.3 ± 4.1	0.290
Smoking habits (% At least one parent smokes)	55.6	52.2	52.2	57.1	0.340
Physical inactivity status (% None of the parents is physically active)	23.8	23.6	26.6	31.0	0.030
Away from the Mediterranean Diet (%)	50.1	40.7	47.7	65.7	<0.001
Stressors					
Number of stressors (%)					
None	12.9	13.7	13.0	11.9	0.015
1–2 stressors	51.7	54.7	52.4	47.4	
≥3 stressors	35.4	31.6	34.6	40.7	
Sources of stress					
Parental expectations (% yes)	36.7	33.7	36.5	41.0	0.023
Teachers’ expectations (% yes)	47.2	43.5	47.5	51.1	0.021
School performance (% yes)	63.1	61.5	63.4	65.0	0.272
Busy schedule of extracurricular activities (% yes)	44.2	39.3	39.2	39.1	0.772
Pressure from classmates/friends (% yes)	15.8	15.7	12.2	21.2	0.038

Notes: Data are presented in the form of mean values ± standard deviation in case of continuous characteristics, and in the form of relative frequencies (%) in case of categorical characteristics; *p*-value was derived from One-way Analysis of Variance and the Pearson’s Chi-square test, in case of continuous and categorical characteristics, respectively; Parental level of adherence to the Mediterranean Diet was estimated through the MedDietScore, and those scoring ≤25 units, were classified as being Away from the Mediterranean Diet; KIDMED score ranges from −4 to 12, where lower scores indicate low adherence to the Mediterranean Diet while higher scores, high adherence to the Mediterranean Diet (≤3, very-low-quality diet; 4–7, need to improve the food pattern to adjust it to the Mediterranean one; ≥8, optimal Mediterranean Diet).

**Table 2:** Results from linear regression models evaluating the association between the N = 1716 children’s UPB consumption (i.e., High vs. Low, High vs. Moderate, Moderate vs. Low) and their BMI (outcome).

Dependent Variable: Body Mass Index (BMI; in kg/m^2^)							
	Model 1 B ± SE, *p*	Model 2 B ± SE, *p*	Model 3 B ± SE, *p*	Model 4 B ± SE, *p*	Model 5 B ± SE, *p*	Model 6 B ± SE, *p*	Model 7 B ± SE, *p*
High vs. Low UPB	0.47 ± 0.21, 0.030	0.43 ± 0.22, 0.049	0.38 ± 0.22, 0.090	0.50 ± 0.28, 0.070	0.49 ± 0.28, 0.090	0.73 ± 0.29, 0.010	0.89 ± 0.37, 0.020
High vs. Moderate UPB	0.74 ± 0.21, <0.001	0.70 ± 0.21, 0.001	0.68 ± 0.21, 0.001	0.72 ± 0.25, 0.004	0.71 ± 0.25, 0.006	0.77 ± 0.26, 0.004	0.69 ± 0.32, 0.030
Moderate vs. Low UPB	−0.27 ± 0.19, 0.150	−0.28 ± 0.19, 0.140	−0.34 ± 0.19, 0.080	−0.25 ± 0.23, 0.280	−0.28 ± 0.23, 0.220	−0.11 ± 0.24, 0.630	−0.09 ± 0.29, 0.740

Notes: Results are presented in the form of beta-coefficients ± SE: Standard errors and *p*-value. Model 1: adjusted for age, sex; Model 2: Model 1+ Children’s level of adherence to the Mediterranean Diet; Model 3: Model 2+ Children’s physical activity status; Model 4: Model 3+ Parental BMI; Model 5: Model 4+ Parental educational level; Model 6: Model 5+ Income status; Model 7: Model 6+ Parental level of adherence to the Mediterranean Diet; BMI: Body Mass Index; UPB: ultra-processed beverages; Parental level of adherence to the Mediterranean Diet was estimated through the MedDietScore, and those scoring ≤25 units were classified as being Away from the Mediterranean Diet; KIDMED score ranges from –4 to 12, where lower scores indicate low adherence to the Mediterranean Diet while higher scores, high adherence to the Mediterranean Diet (≤3, very-low-quality diet; 4–7, need to improve the food pattern to adjust it to the Mediterranean one; ≥8, optimal Mediterranean Diet).

**Table 3:** Results from logistic regression analysis evaluating the association between the preadolescents’ UPB consumption and the sources of stress.

OR (95% CI)	High vs. Low UPB Consumption	Moderate vs. Low UPB Consumption	High vs. Moderate UPB Consumption
Number of stressors (Per 1 stressor increment)	1.11 (1.01, 1.24) **	1.04 (0.95, 1.15)	1.09 (0.99, 1.20) *
Source of stress			
Parental expectations	1.32 (1.01, 1.73) **	1.13 (0.88, 1.44)	1.22 (0.94, 1.57)
Teachers’ expectations	1.33 (1.03, 1.73) **	1.18 (0.93, 1.50)	1.14 (0.89, 1.47)
School performance	1.13 (0.86, 1.47)	1.08 (0.85, 1.38)	1.06 (0.82, 1.37)
Busy schedule of extracurricular activities	1.05 (0.81, 1.37)	1.02 (0.81, 1.30)	1.01 (0.78, 1.30)
Pressure from classmates/friends	1.43 (1.02, 2.02) **	1.32 (0.93, 1.89)	1.91 (1.36, 2.69) ***
Parental + Teachers’ expectations vs. None of the stressors	1.42 (1.02, 2.00) **	1.12 (0.75, 1.69)	1.17 (0.75, 1.81)
Parental expectations + Pressure from classmates/friends vs. None of the stressors	1.99 (1.11, 3.59) **	1.15 (0.64, 2.08)	2.37 (1.34, 4.20) **
Teachers’ expectations + Pressure from classmates/friends vs. None of the stressors	1.63 (1.03, 2.88) **	1.27 (0.72, 2.22)	2.18 (1.24, 3.82) **
Parental + Teacher’s expectations + Pressure from classmates/friends vs. None of the stressors	1.93 (1.01, 3.67) **	1.15 (0.60, 2.22)	2.32 (1.24, 4.36) **
All stressors vs. None of the stressors	2.25 (0.97, 5.19) *	1.15 (0.47, 2.86)	2.84 (1.25, 6.46) **

Notes: *** *p* < 0.001, ** *p* < 0.05, * *p* < 0.10; The results are presented in the form of Odds Ratio (OR) and 95% Confidence Interval (95% CI); The results are adjusted for both children’s (age, sex, level of adherence to the Mediterranean Diet, Physical activity level) and their parents’ characteristics (Parental Body Mass Index, educational level, level of adherence to the Mediterranean Diet, income status); UPB:ultra-processed beverages (soft and flavored drinks); Parental level of adherence to the Mediterranean Diet was estimated through the MedDietScore, and those scoring ≤25 units, were classified as being Away from the Mediterranean Diet; KIDMED score ranges from −4 to 12, where lower scores indicate low adherence to the Mediterranean Diet while higher scores, high adherence to the Mediterranean Diet (≤3, very-low-quality diet; 4–7, need to improve the food pattern to adjust it to the Mediterranean one; ≥8, optimal Mediterranean Diet).

**Table 4:** Results from logistic regression analysis evaluating the association between the preadolescents’ frequency of chocolate milk consumption and the sources of stress.

OR (95% CI)	High vs. Low UPB Consumption	Moderate vs. Low UPB Consumption	High vs. Moderate UPB Consumption
Number of stressors (Per 1 stressor increment)	1.16 (0.99, 1.37) *	1.03 (0.95, 1.13)	1.13 (0.95, 1.35)
Source of stress			
Parental expectations	1.75 (1.13, 2.69) **	0.92 (0.73, 1.16)	1.90 (1.21, 2.98) **
Teachers’ expectations	1.06 (0.69, 1.64)	1.24 (0.99, 1.55) *	0.86 (0.55, 1.35)
School performance	1.03 (0.66, 1.56)	1.24 (0.98, 1.56) *	0.82 (0.52, 1.31)
Busy schedule of extracurricular activities	1.08 (0.70, 1.66)	0.92 (0.74, 1.15)	1.15 (0.73, 1.81)
Pressure from classmates/friends	1.89 (1.14, 3.15) **	0.96 (0.70, 1.32)	1.98 (1.16, 3.38) **
Parental + Teachers’ expectations vs. None of the stressors	1.28 (0.63, 2.60)	1.11 (0.75, 1.65)	1.15 (0.54, 2.43)
Parental expectations + Pressure from classmates/friends vs. None of the stressors	2.59 (1.17, 5.73) **	0.99 (0.58, 1.71)	2.58 (1.09, 6.11) **
Teachers’ expectations + Pressure from classmates/friends vs. None of the stressors	1.98 (0.86, 4.56)	1.18 (0.71, 1.97)	1.58 (0.65, 3.83)
Parental + Teacher’s expectations + Pressure from classmates/friends vs. None of the stressors	2.90 (1.24, 6.78) **	1.08 (0.59, 1.96)	2.61 (1.04, 6.57) **
All stressors vs. None of the stressors	4.54 (1.73, 11.91) **	1.00 (0.44, 2.27)	4.38 (1.47, 12.99) **

Notes: ** *p* < 0.05, * *p* < 0.10; The results are presented in the form of Odds Ratio (OR) and 95% Confidence Interval (95% CI); The results are adjusted for both children’s (age, sex, level of adherence to the Mediterranean Diet, Physical activity level) and their parents’ characteristics (Parental Body Mass Index, educational level, level of adherence to the Mediterranean Diet, income status); UPB: soft and flavored drink; Parental level of adherence to the Mediterranean Diet was estimated through the MedDietScore, and those scoring ≤25 units, were classified as being Away from the Mediterranean Diet; KIDMED score ranges from −4 to 12, where lower scores indicate low adherence to the Mediterranean Diet while higher scores, high adherence to the Mediterranean Diet (≤3, very-low-quality diet; 4–7, need to improve the food pattern to adjust it to the Mediterranean one; ≥8, optimal Mediterranean Diet).

## Data Availability

Data are available upon request. The data are not publicly available due to privacy or ethical restrictions.
